# Trapped by debt: an ethnographic study of medical indebtedness and hospital detention in the Fundong Health District, Cameroon

**DOI:** 10.3389/fpubh.2025.1602798

**Published:** 2025-08-26

**Authors:** Asahngwa Constantine Tanywe, Ngambouk Vitalis Pemunta, Vidarah Nimar, Cybel Nji Angwe, Mathias Alubafi Fubah, Maurine Ekun Nyok, Tom Obara Bosire, Nguyen Ngoc Bich Tram, Brendabell Ebanga Njee, Womma Habiba Hira

**Affiliations:** ^1^Department of Anthropology, University of Yaounde I, Yaounde, Cameroon; ^2^Cameroon Center for Evidence Based Health Care, Yaounde, Cameroon; ^3^Department of Sociology, Covenant University, Ota, Nigeria; ^4^Department of Global Health, University of Gothenburg, Gothenburg, Sweden; ^5^Human Sciences Research Council, Division of Developmental, Capable, and Ethical State (DCES), Pretoria, Gauteng, South Africa; ^6^Research Associate Centre for Gender and Africa Studies, University of the Free State, Bloemfontein, South Africa; ^7^Department of Political Science, University of Hradec Králové, Hradec Králové, Czechia; ^8^Department of Political Science, University of Glasgow, Glasgow, United Kingdom; ^9^Budapest Business School, Budapest, Hungary; ^10^African Public Health Organization, Minnesota, MN, United States; ^11^Department of Global Health, School of Public Health and Community Medicine, University of Gothenburg, Västra Götaland County, Gothenburg, Sweden

**Keywords:** medical indebtedness, hospital detention, Cameroon, universal health coverage, healthcare financing, vulnerable populations

## Abstract

**Background:**

This study investigates the structural and socio-cultural drivers of medical indebtedness and hospital detention due to unpaid healthcare bills in the Fundong Health District, Cameroon. It explores how poverty, institutional shortcomings, and cultural beliefs converge to exacerbate patients’ financial vulnerability and delay access to care.

**Methods:**

A qualitative anthropological approach was employed between February and November 2022, combining 34 in-depth interviews with extended ethnographic observation in hospital wards, billing offices, and family waiting areas. Data were analyzed using iterative grounded theory methods, including open, axial, and selective coding of interview transcripts, focus group discussions, and field notes. This methodology allowed for a nuanced understanding of how debt and detention are experienced and perpetuated. All data were transcribed, manually coded, and analyzed using NVivo 14 software to identify recurring themes related to hospital detention.

**Results:**

The findings show that medical indebtedness is driven by poverty, lack of health insurance, and limited social support. Institutional factors—including underfunded healthcare infrastructure and high user fees—compound these vulnerabilities. Cultural norms, such as beliefs discouraging financial preparation for illness, further heighten exposure to risk. The practice of hospital detention, though largely undocumented, imposes severe physical, emotional, and financial burdens, prompting some to delay care or adopt harmful coping mechanisms.

**Conclusion/policy implications:**

Addressing medical debt and hospital detention requires a multifaceted policy response. Recommendations include eliminating maternal user fees, expanding health insurance coverage for vulnerable populations, protecting hospital-based social assistance, and replacing detention with legal safeguards and social mediation. Additionally, culturally sensitive financial literacy and mental health support programs are vital. Long-term investment in health infrastructure and governance is essential to reduce out-of-pocket spending and ensure equitable, rights-based healthcare access.

## 1 Background

Universal Health Coverage (UHC), enshrined in Sustainable Development Goal (SDG) 3.8, aims to ensure financial risk protection and universal access to quality healthcare services and medicines (1). Yet, this vision remains largely unmet in African health systems, particularly among economically vulnerable populations (1, 2). Despite political commitment, progress is hampered by poverty, weak national fiscal capacity, overreliance on out-of-pocket payments, fragmented financing mechanisms, and underdeveloped insurance schemes (1–8).

Systemic health financing failures in many African countries have resulted in the denial of care for patients unable to pay and, more disturbingly, the widespread practice of hospital detention—defined as “the refusal to release either living patients after they have been medically discharged or the bodies of deceased patients when families cannot afford to pay hospital bills” (p. 1) (9–11). These ethically and legally problematic practices highlight critical gaps in financial risk protection, directly undermining the principles of UHC and calling for urgent reform to eliminate punitive, informal cost-recovery measures.

Hospital detention has been documented in 46 out of 195 countries worldwide—accounting for 24%—with the highest concentration of reported cases occurring in Africa and Asia (10–12). Detentions often exceed a month and disproportionately affect vulnerable groups in public hospitals. Despite growing concern, systematic academic research remains limited. Legal ambiguity and state denial of the practice have left journalistic accounts as the primary source of information.

In Africa, documented cases exist in Cameroon (9), Burundi (12), the Democratic Republic of Congo (13), Kenya (14–17), Nigeria (18–21), Tanzania (22), and Uganda (23). However, scholarly engagement with the issue is sparse. In Cameroon, only one academic study has examined the lived experiences of detained patients (9), leaving significant gaps in understanding the drivers of medical indebtedness and the strategies patients use to prevent or cope with detention.

This study investigates the socio-economic, cultural, and structural drivers of medical indebtedness and hospital detention in Cameroon, employing qualitative methods—including ethnographic fieldwork and in-depth interviews—to center the lived experiences of patients and healthcare workers. It further examines community coping mechanisms and offers policy recommendations to advance healthcare equity, safeguard patient rights, and support the realization of UHC in Cameroon and comparable contexts.

## 2 Study setting

This study investigates the economic burden of hospital detention in the Fundong Health District, located in the conflict-affected North West Region of Cameroon. As a peripheral unit within Cameroon’s three-tier healthcare system, Fundong is responsible for frontline service delivery in a region characterized by chronic poverty, high unemployment, and ongoing armed conflict—factors that significantly exacerbate barriers to healthcare access in the absence of robust financial protection mechanisms.

Cameroon’s health system is structured across three administrative levels: the central level, which defines national policy and governance; regional delegations, tasked with technical oversight; and the peripheral level, where health districts like Fundong provide direct care to communities. In this context, Fundong Health District illustrates how systemic issues—including weak social safety nets and fragmented health financing—interact to deepen economic vulnerability, entrench medical debt, and contribute to the widespread phenomenon of hospital detention.

Although the national healthcare system is formally organized around a referral model—where patients are expected to move from primary care facilities to district, regional, and central hospitals based on case complexity—this pathway is rarely linear in practice. In Fundong, therapeutic decision-making is shaped not only by disease severity but also by cultural understandings of illness and limited financial means. As a result, patients frequently initiate care with home remedies, consult traditional healers, or alternate between biomedical and indigenous practices.

Further complicating care-seeking trajectories are persistent deficiencies in service quality, entrenched mistrust in public institutions, and the absence of effective social protection mechanisms. These structural shortcomings have prompted some individuals to bypass public healthcare facilities entirely, opting instead for costlier private or mission-run hospitals, or in some cases, seeking treatment abroad. Such patterns underscore a deeper erosion of public confidence in domestic health systems and highlight the urgent need for comprehensive reforms to restore trust, improve service delivery, and expand equitable access to quality care (24).

Such care-seeking choices, while aimed at improving health outcomes, regularly heighten financial strain. The cumulative costs associated with navigating diverse and parallel care systems contribute significantly to medical indebtedness. For those unable to pay, hospital detention emerges as a common and punitive consequence. These deviations from the intended referral structure—rooted in economic hardship, cultural beliefs, and systemic failures—underscore the intertwined nature of debt and detention in under-resourced and marginalized areas like Fundong.

In an attempt to address these inequities, the Cameroonian government has implemented exemption policies, including free malaria treatment for children under five and antiretroviral therapy (ART) for people living with HIV (PLHIV). While these initiatives, introduced in 2007 and expanded in 2020 to eliminate direct payments for PLHIV, signal a commitment to financial protection, their impact remains limited due to fragmented implementation and persistent socioeconomic disparities (25–2).

Health financing in Cameroon is dominated by out-of-pocket (OOP) payments, which account for the majority of healthcare expenditure and are widely regarded as regressive and impoverishing (25). Only about 2% of Cameroonians are covered by any form of social health protection (26) underscoring the slow pace toward UHC. The Fundong Health District exemplifies these systemic challenges, with most households relying on direct payments for care—frequently leading to medical debt and hospital detention when patients are unable to settle bills.

In line with Cameroon’s decentralized health financing strategy, Fundong implemented a Community-Based Health Insurance (CBHI) scheme aimed at reducing the burden of OOP expenses. While specific premium data for Fundong are scarce, comparable schemes in Cameroon typically charge around 9,000 XAF (approx. 15 USD) annually per household, with premiums adjusted for income and family size (27, 28). We were given to understand that the CBHI coverage usually includes outpatient consultations, maternal and child healthcare, hospitalizations, and common illnesses, though actual benefits vary depending on facility agreements (28). These schemes typically operate on a co-payment model where the scheme covers 80% of the costs and members are responsible for the remaining 20%. Despite its initial promise, Fundong’s Community-Based Health Insurance (CBHI) initiative has faltered—particularly under the strain of the ongoing Anglophone crisis, which began in 2016. This conflict, rooted in the political and socio-economic marginalization of Cameroon’s English-speaking regions, has led to widespread instability and the disruption of public services. This unrest has contributed to administrative breakdowns, declining enrollment, and a loss of trust in the system. As a result, structural challenges—such as limited resources and partial insurance coverage—continue to undermine the program’s ability to fully support patients, increasing the risk of high out-of-pocket costs and long-term medical debt. In parallel, the Ministry of Social Affairs (MINAS) supports indigent patients through financial aid, social protection programs, and partnerships with faith-based organizations such as the Cameroon Baptist Convention Health Services. Between 2007 and 2012, over 1.3 billion FCFA was allocated for vulnerable populations, yet bureaucratic inefficiencies and low public awareness have constrained its reach (29–31).

### 2.1 Fundong Health District

The Fundong Health District, located in Boyo Division of Cameroon’s North West Region, encompasses four subdivisions—Fundong, Njinikom, Belo, and Bum—and operates mainly through three local councils: Fundong, Njinikom, and Belo, with partial jurisdiction in Konene Health Area of Bum. Its estimated population of 146,162, primarily from the Kom ethnic group, depends largely on subsistence agriculture, small-scale trade, and palm wine tapping—a reflection of informal labor patterns and economic precarity (32).

Healthcare in the district is delivered through a network of 27 facilities comprising public, private for-profit, and private not-for-profit (mostly faith-based) providers. Of these, 18 are public, 2 are private for-profit, and 7 are faith-based, with the latter disproportionately located in rural, underserved areas. Key institutions include the Fundong District Hospital, St. Martin de Porres Catholic General Hospital in Njinikom, the Akeh Baptist Health Center, and Ilung’s two faith-based clinics (32).

**Figure 1:**
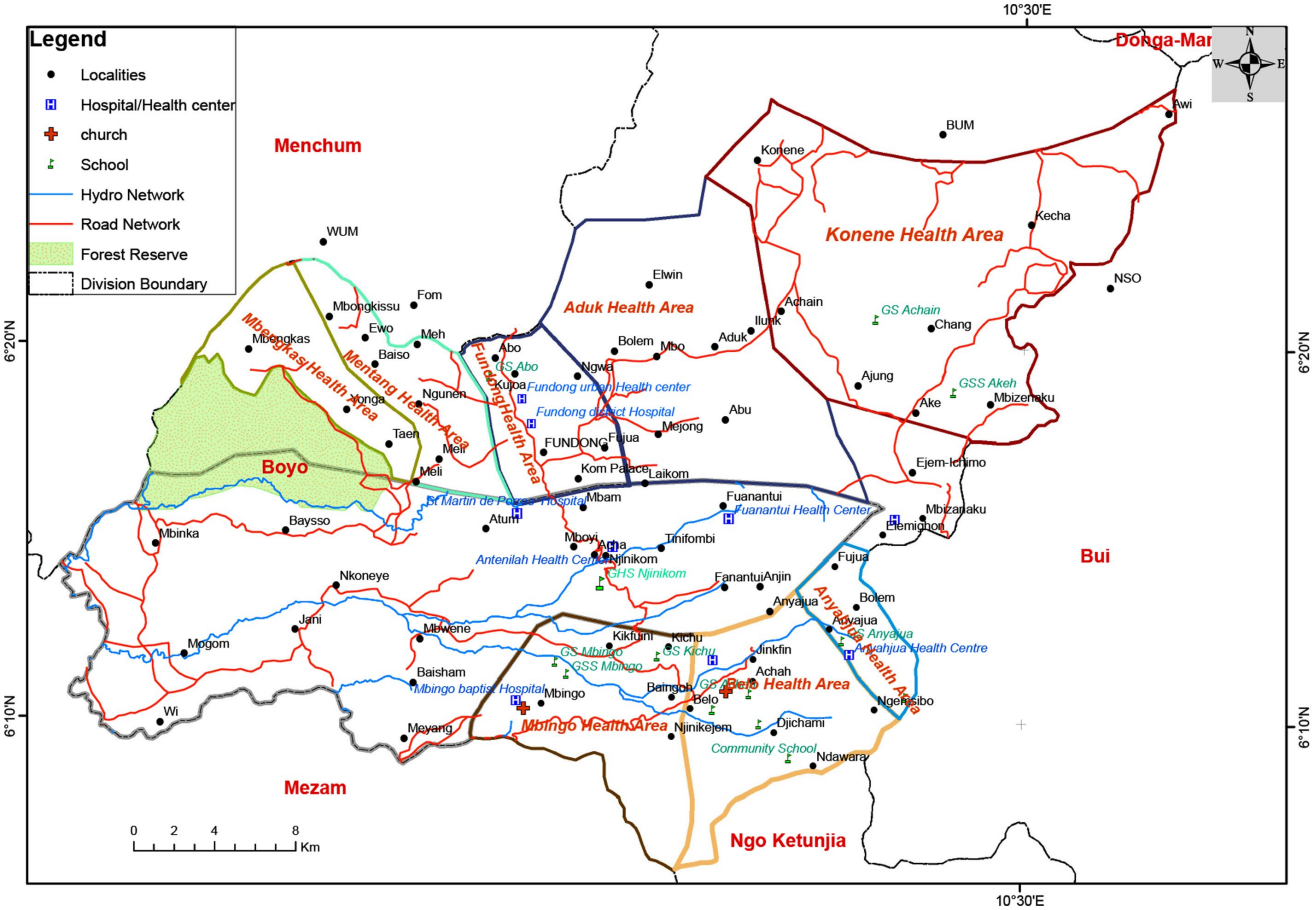
Map of Fundong Health District showing health areas and hospitals. Source: Provincial delegation of public health, Northwest Region.

Faith-based providers are not merely supplemental but constitute an integral component of healthcare delivery in Fundong, frequently perceived as offering more compassionate care. This perception fosters religiously mediated expectations—patients anticipate reduced fees or free care as reciprocity for their previous contributions to religious communities. However, most mission hospitals continue to require payment for services, and in the absence of transparent subsidy mechanisms, patients remain at risk of accumulating debt and facing detention for non-payment (Figure 1).

Topographically, Fundong’s mountainous terrain and poor road infrastructure compound healthcare access challenges, resulting in delayed or missed treatments and worsening health outcomes. It further compounds the socioeconomic challenges faced by a population largely engaged in subsistence agriculture and informal economic activities (32).

Healthcare financing in Fundong reflects the broader national landscape: a fragmented system characterized by community-based health insurance (CBHI), limited government subsidies, ethno-health schemes, and a heavy reliance on faith-based providers. While CBHI organizations such as the Boyo Mutual Health Organization and the Bamenda Provincial Ecclesiastical Health Assistance provide partial financial protection, they remain underfunded, poorly coordinated, and weakly integrated within the national health framework. The collapse of Fundong’s CBHI scheme—exacerbated by the ongoing Anglophone crisis since 2016—has significantly eroded community trust and disrupted local health financing mechanisms. Within this context, hospital detention has emerged as a systemic response to unpaid bills, particularly within mission hospitals, where it serves as a prevalent form of informal debt enforcement.

Nationally, less than 2% of Cameroonians are covered by formal health insurance, and fee exemption policies—when they exist—are applied inconsistently at best (26, 27). These systemic shortcomings leave families vulnerable to catastrophic health expenditures and have fostered widespread disillusionment with the public healthcare system. As a result, some patients resort to seeking expensive medical treatment abroad, despite the significant financial burden this entails.

Against this backdrop, the Fundong Health District offers a critical case study for examining how individual vulnerability, community expectations, and structural financing deficits intersect to produce—and normalize—the practice of hospital detention. This analysis draws on vulnerability theory and social safety theory to explore how social structures, exposure to risk, and institutional fragilities combine to shape patterns of healthcare access and the financial consequences for individuals and families.

## 3 Methods and theoretical framework

This study employed a qualitative research design, guided by ethnographic and grounded theory approaches, to explore the socio-economic and institutional factors contributing to medical indebtedness and hospital detention in Fundong Health District. The Fundong Health District was purposively selected as the study site due to the observable and documented prevalence of hospital detention—where patients are confined in health facilities as a result of unpaid medical bills. Preliminary field observations, supported by reports from local health workers and community members, indicated that financial insolvency is a recurring barrier to discharge. This made the site particularly suitable for exploring the socio-economic and institutional factors contributing to medical indebtedness through direct observation and ethnographic engagement (9, 11).

Data collection involved a combination of six focus group discussions (FGDs), semi-structured interviews, and prolonged participant observation conducted across hospital wards, billing offices, and family waiting areas. These qualitative methods enabled triangulation across the perspectives of detained patients, caregivers, hospital administrators, healthcare workers, and community stakeholders. This multi-method approach provided both narrative depth and contextual sensitivity, facilitating a comprehensive understanding of participants’ lived experiences with healthcare-related debt and detention.

To refine the research tools and approach, a pilot study was conducted at Njinikom Hospital, a rural, faith-based facility. This phase tested the cultural relevance and methodological rigor of the interview and FGD guides. Based on feedback from hospital staff and participants, questions related to debt were rephrased for greater sensitivity, and rapport-building techniques were strengthened. These revisions ensured ethical alignment and cultural resonance, in line with best practices for cross-cultural qualitative research (29–31).

Participant observation offered valuable insights into the unspoken norms and institutional routines influencing patient and caregiver behavior. Conducted across various hospital spaces, it uncovered subtle yet telling practices—such as the quiet abandonment of personal items like identity cards, clothing, and food—driven by fear of detention. This immersive approach revealed how vulnerability and institutional power are enacted in the everyday dynamics of healthcare settings. Together, these methods supported a multi-perspectival, ethically grounded, and contextually embedded analysis, thereby enhancing the validity, depth, and cultural sensitivity of the study’s findings.

Prior to the main fieldwork, the research team underwent cultural affinity training, working closely with local mediators and healthcare professionals to develop an informed understanding of local communication patterns, kinship structures, and hospital hierarchies. This training enhanced the team’s cultural competence, minimized miscommunication, and increased trustworthiness during data collection (33–41).

The fieldwork proceeded in an iterative and flexible manner, allowing the research design to evolve based on emerging insights and participant feedback. This approach was particularly effective in surfacing subtler dynamics, such as the emotional strain of prolonged hospitalization and the social stigma attached to hospital detention for nonpayment. As researchers with prior experience in health systems and social inequalities in Cameroon, we approached the field with a reflexive awareness of our positionality—as both insiders familiar with the socio-cultural context and outsiders shaped by academic training and institutional affiliations. This dual positionality facilitated trust-building with participants while requiring continual self-reflection to ensure that local narratives and priorities guided the research process.

A purposive sampling strategy was employed to recruit participants with direct or indirect experience of medical indebtedness or hospital detention. Key stakeholder groups included indebted patients, individuals who had been detained in hospitals, affected family members, healthcare providers, and community leaders familiar with local health financing issues. This approach ensured the collection of rich and relevant data for the study.

To ensure diverse socio-economic representation, participants were recruited from multiple settings within the Fundong Health District, including urban and rural areas, as well as from different income levels and occupations. Local health facilities and community organizations assisted in identifying eligible individuals through referral and snowball techniques, emphasizing inclusion of marginalized groups typically underrepresented in health research.

Screening criteria included willingness to discuss financial and healthcare experiences and being residents within the district. This approach allowed the study to capture a broad spectrum of perspectives on the financial barriers to healthcare and the social implications of hospital detention, thereby enhancing the depth and validity of the findings.

The study’s analytical framework combines vulnerability theory and social safety theory to examine the complex interplay between structural factors and individual experiences contributing to healthcare-related financial hardship. Originally developed in disaster and environmental research, vulnerability theory is applied here to highlight how systemic inequities—such as poverty, gendered caregiving roles, and social marginalization—increase individuals’ risk of incurring medical debt (42–47). Challenging the conventional assumption that debt leads to vulnerability, this study argues that existing vulnerabilities are the primary drivers predisposing individuals to medical indebtedness. In this framework, vulnerability is positioned as the antecedent rather than the outcome of debt.

Importantly, the decision to reverse the causal relationship—from viewing vulnerability as a result of debt to recognizing it as a precursor—emerged inductively through the study’s flexible and iterative design. This adaptive process enabled the theoretical framework to evolve in response to empirical findings, rather than being fixed before data collection.

Complementing this, social safety theory sheds light on how individuals perceive and manage threats to their well-being in medical settings (48–50). The study privileges subjective experiences of insecurity—such as fear of detention, social shame, and emotional distress—over more easily quantifiable markers like discharge policies or debt thresholds. Particular attention is given to informal coping mechanisms such as reliance on kinship networks, pooling of resources, and community-based solidarity, which, though adaptive, may unintentionally entrench cycles of indebtedness and social dependence.

By embedding hospital detention within wider discourses of structural vulnerability, informal economies of care, and patient agency, this study contributes to growing empirical and theoretical work on the social production of financial hardship in healthcare—especially in low- and middle-income settings. Furthermore, this approach draws on multi-scalar ethnographic methods that blend narrative, observational, and institutional data to provide a richer understanding of the patient experience.

Data analysis followed an iterative coding process informed by both ethnographic and grounded theory methodologies. Interview and focus group transcripts, along with field notes from participant observation, were subjected to open, axial, and selective coding to uncover recurring patterns, relational dynamics, and emergent themes. This inductive approach enabled a contextually grounded and multi-perspectival interpretation of how medical debt and detention are produced and sustained within the Fundong Health District. All data were transcribed, manually coded, and analyzed using NVivo 14 software, facilitating a nuanced understanding of the lived experiences and systemic perpetuation of hospital detention.

By integrating multiple qualitative methods with a focus on reflexivity and cultural sensitivity, the study offers valuable insights into the interactions between healthcare systems, patient coping strategies, and institutional policies.

## 4 Results

A total of 34 participants were included in the study: 8 detained patients and 26 hospital staff members. The hospital staff comprised 2 managers, 2 billing clerks, 8 receiving nurses, 6 ward charge nurses, 2 social workers, and 6 security personnel. Participants’ ages ranged from 20 to 50 years, with 14 individuals (41.2%) falling between 31 and 40 years old. The overall sample was predominantly female. This diverse composition and methodological triangulation enabled the development of grounded, context-sensitive insights into institutional practices and lived experiences related to hospital detention and medical debt.

Among the 8 detained patients, 5 were women (62.5%) and 3 were men (37.5%). In terms of education, 5 patients (62.5%) had completed primary education and 3 (37.5%) had completed secondary education. None of the detained patients had attained tertiary education or reported having no formal education.

Regarding marital status, 5 patients (62.5%) were single and 3 (37.5%) were married. All 8 patients (100%) identified as self-employed, with no participants engaged in public or private sector employment.

The duration of detention varied: 3 patients (37.5%) were held for 1–4 weeks, another 3 (37.5%) for 5–8 weeks, and 2 patients (25%) for periods between 9 and 52 weeks. No participant reported being detained for more than a year. These socio-demographic characteristics and related experiences with medical indebtedness are summarized in Table 1.

**Table 1:** Socio-demographic characteristics of detained insolvent patients (*n* = 8).

Demographic categories	Subcategories	Number of Participants (*n* = 8)	Percentage (%)
Age	20–30	2	25.0%
	31–40	4	50.0%
	41–50	2	25.0%
	51 and above	–	0.0%
Sex	Male	3	37.5%
	Female	5	62.5%
Level of education	Never Attended	–	0.0%
	Primary	5	62.5%
	Secondary	3	37.5%
	Tertiary	–	0.0%
Marital status	Married	3	37.5%
	Single	5	62.5%
Employment status	Public	–	0.0%
	Private	–	0.0%
	Self-Employed	8	100.0%
Length of detention	1–4 weeks	3	37.5%
	5–8 weeks	3	37.5%
	9–52 weeks	2	25.0%
	>52 weeks	–	0.0%

Source: Fieldwork (The length of detention” refers specifically to the number of days a patient remains hospitalized beyond the official discharge date as determined by a health professional).

The disproportionate representation between detained patients (*n* = 8) and hospital staff (*n* = 26) reflects the methodological and ethical complexities inherent in recruiting detained individuals for qualitative research. Although the study sought to incorporate balanced viewpoints from both groups, the recruitment of patients was constrained by institutional approval processes, confidentiality considerations, and limited access. Despite these challenges, the inclusion of hospital staff across administrative, clinical, and security roles provided a multifaceted institutional lens that effectively complemented patient narratives. Together, these perspectives yield a more holistic understanding of hospital detention, illustrating the intersection of structural conditions and individual agency.

Among the 26 staff participants, the majority were women (69.2%, *n* = 18), with men comprising 30.8% (*n* = 8). Most staff were aged between 31 and 50 years (73.1%, *n* = 19), and all were employed in the private healthcare sector, with no respondents indicating employment in public institutions or self-employment.

With respect to educational attainment, 69.2% (*n* = 18) had completed primary education and 30.8% (*n* = 8) had reached the secondary level. No participants reported either a lack of formal education or possession of tertiary qualifications. In terms of marital status, a substantial majority were married (84.6%, *n* = 22), while the remainder were single (15.4%, *n* = 4).

This demographic profile—detailed in Table 2—characterizes a workforce that is predominantly female, middle-aged, privately employed, and moderately educated. These attributes provide essential context for interpreting staff perceptions of institutional practices, particularly in relation to hospital detention and the management of patient debt.

**Table 2:** Socio-demographic characteristics of health personnel in the study (*n* = 26).

Demographic category	Subcategories	Number of participants (*n* = 26)	Percentage (%)
Age	20–30	5	19.2%
	31–40	11	42.3%
	41–50	8	30.8%
	51 and above	2	7.7%
Sex	Male	8	30.8%
	Female	18	69.2%
Level of education	Never Attended	–	0.0%
	Primary	18	69.2%
	Secondary	8	30.8%
	Tertiary	–	0.0%
Marital status	Married	22	84.6%
	Single	4	15.4%
Employment status	Public	–	0.0%
	Private	26	100.0%
	Self-Employed	–	0.0%

Source: Fieldwork.

By incorporating the perspectives of both detained patients and healthcare personnel, the study provides a nuanced and comprehensive understanding of hospital detention and medical indebtedness. This dual perspective sheds light on systemic challenges—including financial barriers, informational gaps, and socio-cultural influences—that shape patients’ vulnerability to debt and prolonged hospitalization.

Notably, the absence of participants in specific socio-demographic categories—such as no detained patients having attained tertiary education, no public sector representation among healthcare staff, and no hospital staff members lacking basic formal education—is indicated as “---” in the tables. These omissions underscore structural inequalities and institutional patterns that significantly impact access to care and the lived experiences of both patients and providers.

### 4.1 Thematic summary

This study identified three interrelated levels of influence—interpersonal, organizational, and community—shaping the experience of medical indebtedness and hospital detention. Each level revealed distinct but interconnected themes, highlighting the complex drivers behind patient vulnerability and systemic failure. Table 3 summarizes these themes, based on the prominence they received in participant narratives.

**Table 3:** Summary of thematic factors contributing to medical indebtedness and hospital detention.

Level of influence	Key themes	Illustrative quotes (with Speaker Profile)
Interpersonal	Dependence on family for support	“I cannot pay my bill… I have no family support.” (Patient)
	Miscommunication about treatment costs	“They never told me how much it would cost.” (Patient)
	Cultural beliefs discouraging savings	“We keep money for good things. Sickness is bad.” (Community Member)
	Family and religious norms shaping behavior	“As Muslims, we do not keep money for sickness.” (Community Member)
Organizational	Delayed disclosure of hospital fees	“Patients often come in without knowing the cost….” (Health Provider)
	Inability to pay due to absence of insurance coverage	“There is no insurance in this area, so patients must pay everything themselves.” (Health Provider)
	Variation in payment policies across hospital types	“In this hospital, we save lives with or without money.” (Hospital Administrator)
Community	Community-based support through religious institutions	“As a church, we have to help them….” (Religious Leader)
	Inadequate reach of community insurance	“We have a community-based insurance scheme, but many cannot afford it or do not know how it works.” (Community Health Worker)
	Norms expecting care regardless of payment	“For those who do not have money, treatment continues.” (Health Provider)
	Informal negotiation of fees	Theme noted across various accounts

Source: Field notes.

This thematic structure offers a nuanced understanding of the layered factors that sustain financial hardship and hospital detention. Participants’ accounts reveal how miscommunication, gaps in insurance coverage, institutional inconsistencies, and deeply rooted cultural norms converge to shape care experiences. These interconnected influences underscore the need for context-sensitive interventions that address both systemic inequities and the localized social expectations governing access to healthcare.

### 4.2 Multilevel drivers of medical indebtedness and hospital detention

This section provides an in-depth analysis of the interconnected interpersonal, organizational, and community-level factors that contribute to medical indebtedness and hospital detention. Drawing from the thematic summary and grounded in participant narratives, the discussion highlights how financial hardship is not merely an individual issue but a product of broader systemic, cultural, and institutional dynamics.

At the interpersonal level, challenges include miscommunication about treatment costs, cultural norms that discourage saving for illness, and reliance on family networks for financial support. These elements heighten vulnerability and delay payment, as expressed by one patient: *“I cannot pay my bill… I have no family support.”*

Organizational factors reflect limited or absent health insurance options, inconsistent billing practices, and policy discrepancies between public and private hospitals. These institutional inconsistencies can either mitigate or exacerbate the risk of detention, as illustrated by a provider’s comment: *“In this hospital, we save lives with or without money.”*

At the community level, factors include poor awareness of support services, limited access to community-based insurance schemes, and widespread social expectations that care should be provided irrespective of a patient’s financial capacity. One participant noted, “We have a community-based insurance scheme…” — though such initiatives frequently suffer from insufficient coverage and a lack of clear communication.

Together, these overlapping forces shape the lived experience of medical debt and detention. This analysis underscores the need for context-sensitive reforms that address not only structural inequalities but also localized socio-cultural dynamics influencing access to care.

#### 4.2.1 Interpersonal factors

At the interpersonal level, family dynamics and social networks play crucial roles in shaping health-seeking behaviors and the financial pressures faced by patients. In resource-constrained settings, family members frequently serve as primary caregivers and financial supporters, particularly when patients lack the means to afford healthcare services. Reliance on family for financial support can either mitigate or intensify the financial burden associated with medical costs. One respondent from Facility A (male, 42 years old) shared:

*“I am here because I cannot pay my bill. I earn 10,000 CFA* (approximately $16.50 USD) *per month, and I have nothing left after covering basic needs. To pay my 400,000 CFA* (approximately $660 USD) *bill, I would need to work here for at least 4 years. I sleep on the veranda, outside on a mattress. I have no family support to help cover these costs. If I get sick, I’ll consult, receive treatment, and the cost will just be added to my bill.”*

This statement underscores how poverty and lack of family support contribute to escalating medical debt. Patients unable to afford treatment frequently delay or forgo care and may be detained in hospitals due to unpaid bills. These experiences highlight the burden of unaffordable services, particularly in conflict-affected settings such as Northwest Cameroon. Although not always explicitly stated, financial inaccessibility emerged as a prominent theme in participants’ accounts.

A nurse (42 years old) from Hospital A recounted,

*“I have witnessed numerous cases where adolescent girls are left to fend for themselves after becoming pregnant. These minors often lack the financial means to cover medical expenses. In many instances, complications during childbirth necessitate cesarean sections, which significantly increase hospital costs. As a result, they are sometimes forced to remain in the hospital until the outstanding bills are settled, thereby further escalating the financial burden associated with childbirth and hospitalization.”*

This testimony reflects a broader structural issue within healthcare systems in resource-limited settings, where social and economic vulnerabilities intersect with inadequate health financing mechanisms. The abandonment of adolescent girls—both socially and financially—exacerbates their exposure to medical and economic risks. Cesarean sections, while medically necessary, frequently result in high out-of-pocket expenses, especially in contexts where health insurance coverage is minimal or nonexistent.

Cultural beliefs about illness also significantly influence how individuals prepare for healthcare costs. A female participant from Facility B (31 years old) explained:

*“We keep money to be used for good things. Sickness is very bad. Why should one keep money for it? If you do that, it means you are inviting sickness to your home, which is not a good thing to do.”*

The reluctance to save for future illness stems not merely from economic constraints but from deeply rooted cultural and spiritual beliefs. Several participants perceived saving money specifically for potential illness as culturally taboo, associating it with the risk of “inviting” misfortune or symbolically calling illness into existence. This form of magical thinking, observed in various cultural contexts, suggests that planning for negative events may cause them to occur. While individuals frequently save for socially celebrated milestones such as weddings, education, or housing, these funds are seldom redirected toward healthcare. Doing so may not only disrupt important social obligations but also carry spiritual unease or stigma. Although some families eventually liquidated assets or diverted funds in emergencies, such actions were typically seen as last resorts rather than prudent financial planning. This illustrates that health-related financial behaviors are shaped by a complex interplay of cultural norms, spiritual worldviews, and economic realities.

Another respondent from the same facility (male, 35 years old) added:

*“As Muslims, we do not keep money for sickness. We keep money for good things and not for sickness because it is something bad. This is not acceptable.”*

This comment underscores the influence of religious and cultural beliefs on financial planning for healthcare. The speaker suggests that saving money for illness is unacceptable within their faith, as sickness is viewed as a negative occurrence that should not be anticipated.

#### 4.2.2 Organizational factors

At the organizational level, multiple factors contribute to medical indebtedness. Limited insurance coverage and inadequate communication regarding treatment costs significantly increase the financial burden on patients. Both healthcare staff and patients expressed concern over the high cost of care, particularly in private and faith-based hospitals. The absence of transparent information about treatment expenses frequently results in patients accumulating substantial debt before fully comprehending the financial implications of their healthcare.

One nurse at Facility A (female, 39 years old) explained:

*“Some patients come here without knowing how much their treatment will cost. They expect to pay later, but by the time they realize the amount they owe, they are overwhelmed by the debt. This creates a lot of tension.”*

This comment illustrates the frustration patients experience due to a lack of upfront communication about treatment costs—an omission that generates significant financial uncertainty and, in some cases, leads to hospital detention when patients are unable to meet payment demands.

A hospital administrator from Facility B (male, 50 years old) stated:

*“In this hospital, we save lives with or without money. Our priority is to treat patients, not to turn them away because they cannot pay. However, we are limited by the lack of government funding and insurance options for the poor. This limits our ability to provide sustained care.”*

This statement highlights the moral and ethical dilemmas healthcare providers face in balancing financial sustainability with the imperative to deliver essential healthcare services. The ethical obligation to treat patients regardless of their ability to pay frequently conflicts with the practical challenges of operating within limited financial resources.

#### 4.2.3 Institutional practices and the Erosion of patient dignity

Participant observation within hospital wards revealed subtle yet impactful institutional practices that intensified the experiences of detained patients. These observations, conducted across multiple healthcare facilities in the Fundong Health District, uncovered unspoken but routine behaviors that frequently remained unnoticed during interviews or focus group discussions. One particularly telling pattern was the discreet abandonment of personal items—such as clothing, toiletries, or medical records—by patients who feared being detained for unpaid bills. This act functioned as a quiet but potent expression of resignation and fear, underscoring the extent to which financial vulnerability shaped patient behavior even before formal discharge procedures were initiated.

Moreover, spatial arrangements and verbal interactions within the wards reinforced a hierarchy among patients, distinguishing between those who had paid and those who had not. Detained patients were sometimes placed in less visible corners of the ward or relegated to side rooms, implicitly marking them as financial defaulters. In some instances, hospital staff were observed addressing detained patients with a tone that lacked the empathy typically extended to others—signaling a subtle erosion of dignity linked to indebtedness.

These practices were rarely documented in hospital policies or openly acknowledged by staff, yet they operated as informal mechanisms of control and moral judgment, reflecting institutional attitudes toward poverty and indebtedness. While not always overtly abusive, such interactions had cumulative psychological effects on patients and caregivers, reinforcing feelings of shame, exclusion, and helplessness.

These insights reveal how institutional structures and routines, even when not codified, contribute to the lived realities of hospital detention. They underscore the importance of ethnographic observation in capturing the nuanced ways in which financial precarity is embodied and experienced within healthcare spaces—insights that may be missed through interviews alone. This evidence points to the institutional production of stigma and the systematic erosion of dignity, thereby complicating narratives that portray hospital detention as a purely financial or logistical issue.

#### 4.2.4 Community factors

At the community level, patients frequently depend on informal support networks or community-based insurance schemes to offset medical expenses. However, limited awareness of these available support mechanisms increases patients’ vulnerability to accumulating medical debt. Several respondents reported that community leaders or religious organizations occasionally intervene to negotiate repayment terms or offer financial assistance. As one 40-year-old female patient from Facility A recounted:

*“I did not know there were options to help me pay my bills. Now, I have learned from the nurse that I could get some help from the government. But it was after I had already accumulated over 500,000 CFA in debt. I wish I had known sooner.”*

This statement highlights the gap in knowledge about available financial support systems, suggesting the need for greater awareness and communication about assistance programs that could help prevent patients from falling into debt.

Another participant from Facility B (male, 33 years old) explained:

*“We have a community-based insurance scheme, but it’s not enough to cover all the costs. Still, we have to keep treating those who do not have money. As a church, we help them, especially the most vulnerable members.”*

This quote underscores the critical role of community-based support systems—such as local insurance schemes and religious organizations—in providing financial assistance to patients in need. However, these systems face significant limitations due to resource constraints and coverage gaps, which prevent them from fully addressing the needs of all patients. As a result, a considerable number of individuals remain responsible for substantial out-of-pocket expenses, increasing their risk of medical indebtedness.

The following section reframes medical debt not just as a financial issue, but as a reflection of structural vulnerabilities. Using vulnerability and social safety theories, it highlights how overlapping risks at individual, institutional, and community levels drive outcomes like hospital detention.

## 5 Discussion

Medical debt is not merely a consequence of illness, but a reflection of deeper structural vulnerabilities. Drawing on vulnerability theory and social safety theory, this section interprets medical indebtedness and hospital detention as outcomes of intersecting risks at the individual, institutional, and community levels. The findings reveal that financial hardship arises from a combination of miscommunication about healthcare costs, limited insurance coverage, cultural beliefs discouraging proactive health savings, and low awareness of available support systems. These factors collectively shape patients’ ability to access and afford care. Addressing them requires more transparent cost communication, expanded outreach on financial assistance programs, and culturally sensitive efforts to shift prevailing attitudes toward healthcare planning and savings.

### 5.1 Interpersonal factors

The findings reveal that the type of illness—particularly acute and chronic conditions requiring expensive interventions such as cesarean sections and surgical procedures—is a key driver of financial insolvency. Through the lens of vulnerability theory, these cases underscore how structural and pre-existing vulnerabilities, including poverty, youth, and lack of health insurance, compound the risk of financial harm during medical emergencies [e.g., (12–23, 25, 51, 52)]. A prominent example emerging from the data is teenage pregnancy. Adolescent mothers not only encounter elevated medical risks but are also frequently abandoned by their partners, exacerbating their financial vulnerability. This situation frequently results in indebtedness and, in extreme cases, hospital detention. This pattern has been well documented across several African countries—such as Burundi (12), the Democratic Republic of Congo (14), Kenya (15–17), Nigeria (18–21), Uganda (23), and Tanzania (22)—where patients, particularly those who have undergone cesarean deliveries, are regularly detained in health facilities due to unpaid medical bills (12–21).

Rather than treating indebtedness as the origin of vulnerability, this study aligns with a reverse-causal interpretation—pre-existing vulnerabilities precipitate medical debt, not vice versa (26–28). The caregiving role of families further reinforces this interpretation. While some families successfully pool resources to secure a patient’s discharge, such collective efforts may also exacerbate long-term financial strain, perpetuating a cycle of vulnerability (29–31, 33, 34). The interplay between family caregiving and financial fragility is particularly pronounced in contexts where caregiving responsibilities disproportionately fall on women, whose economic roles are already constrained by broader socio-economic inequalities.

Furthermore, the findings demonstrate how individual circumstances and socio-cultural factors converge to influence healthcare experiences. Patients residing in rural areas, characterized by limited access to financial resources and social networks, exhibited heightened vulnerability to hospital detention. Conversely, urban patients, despite greater access to formal financial mechanisms such as credit and insurance, encountered more complex logistical challenges in obtaining timely care, including prolonged waiting periods and bureaucratic inefficiencies. These observations underscore the significant influence of geographic and socio-economic factors on healthcare access and the attendant risk of medical indebtedness.

### 5.2 Organizational and institutional factors

At the organizational level, the findings highlight how structural limitations—such as fragmented health insurance schemes and underfunded public healthcare—contribute to institutionalized vulnerability. According to vulnerability theory, such institutional weaknesses magnify exposure to risk by failing to buffer individuals from economic shocks (51). The lack of comprehensive health coverage in the study setting left patients exposed to unpredictable costs, exacerbating their financial vulnerability and increasing the likelihood of hospital detention.

The absence of UHC, coupled with inconsistent enforcement of payment and discharge policies, creates a precarious environment for both patients and healthcare providers (33, 34, 42–47). This corresponds with social safety theory, which differentiates between objective safety mechanisms (e.g., payment systems, discharge protocols) and subjective perceptions of security. In the study, patients navigating these systems frequently lacked clarity or confidence in institutional procedures, resulting in fear-driven decisions such as delaying care or seeking alternative therapies (25, 52). The lack of transparent communication and the unpredictability of discharge processes fostered feelings of insecurity, particularly among vulnerable patients burdened by both illness and financial instability.

Institutional behavior reflects diverse logics of care. Faith-based hospitals prioritize treatment over payment, emphasizing moral responsibility and compassion, whereas public hospitals enforce more stringent payment policies. These differing approaches generate distinct forms of vulnerability: financial insecurity stemming from unpaid debts in the former, and medical risk due to delayed or denied care in the latter. Such contradictions demonstrate how institutional frameworks co-produce vulnerability, contingent upon patients’ socioeconomic status. While the faith-based model embodies compassion, it may inadvertently exacerbate financial instability by providing care without clear mechanisms for financial accountability. Conversely, the rigorous payment requirements of public hospitals can lead to refusal of treatment, leaving patients in critical medical need without alternatives.

### 5.3 Community-level and family-based factors

Family and community networks, traditionally regarded as buffers against hardship, occupy an ambivalent position within both vulnerability theory and social safety theory. The study reveals that while kinship structures serve as essential support systems, they remain susceptible to broader economic precarity. Numerous patients reported limited capacity to rely on family assistance due to shared financial hardship or social estrangement, particularly in urban contexts. Among those who did receive support, it was commonly observed that family members themselves incurred debt to facilitate access to treatment (25, 33, 34, 42–47). This illustrates how kinship, rather than functioning as an unequivocal safety net, may contribute to perpetuating cycles of indebtedness when family members are likewise economically vulnerable.

This reflects social safety theory’s emphasis on subjective safety: although family-based interventions may temporarily alleviate distress, they rarely resolve the underlying financial insecurity. Patients experience a fragile sense of safety—both emotional and material—which can easily collapse under sustained economic pressure (25, 52). In such contexts, the role of community support becomes crucial. However, as the study suggests, CBHIs offer only partial protection due to limited integration into national healthcare systems and inconsistent regulation. While CBHIs reflect an awareness of collective risk, their limited reach and financial sustainability constrain their ability to provide substantial relief for the most vulnerable.

Moreover, cultural beliefs influence how individuals perceive and respond to risk. The notion that saving money for health expenses might invite misfortune undermines preventive health financing. Similarly, expectations that faith-based care should be provided free of charge result in insufficient financial planning, thereby increasing the risk of debt and detention. These beliefs represent culturally embedded vulnerabilities that shape risk exposure and coping capacities, consistent with vulnerability theory (51). The interaction between cultural beliefs and financial insecurity produces a precarious environment in which patients may elect to forgo preventive care or rely on informal and less effective treatment modalities.

### 5.4 Structural vulnerabilities and the consequences of medical indebtedness

The prolonged detention of patients due to unpaid hospital bills represents more than a financial or administrative failure—it constitutes a violation of basic human rights and reinforces entrenched cycles of poverty and exclusion. This practice, particularly prevalent in maternal healthcare settings across many African countries, illustrates how gender, age, and economic precarity intersect to create layers of compounded vulnerability [see (51, 52)]. Medical indebtedness is not simply a result of individual inability to pay but rather the outcome of systemic barriers spanning interpersonal misunderstandings, weak institutional frameworks, and inadequate community support.

Patients and families commonly resort to informal coping mechanisms such as early discharge, use of traditional remedies, or negotiation of unofficial payment arrangements. These strategies, intended to prevent further debt or detention, take place in the absence of dependable institutional support. Rooted in both pragmatic economic considerations and the desire to maintain dignity and agency amid systemic disempowerment, these behaviors are influenced by broader socio-cultural norms and constraints (25, 52). While such coping mechanisms may offer temporary relief, they frequently contribute to deteriorating health outcomes. Delaying or avoiding care due to fear of detention can result in disease progression, necessitating more intensive and costly interventions, thereby perpetuating the cycle of medical indebtedness.

Vulnerability theory elucidates this recursive pattern: each failed coping mechanism increases future susceptibility to harm (25, 51, 52). Families already at the edge of economic collapse are pushed deeper into poverty, while the health system’s lack of formal safety nets exacerbates patient suffering. This study’s data reveal that hospital detention and indebtedness are not isolated incidents but embedded within a broader structural landscape of inequality.

Together, these findings highlight the urgent need for context-sensitive policy interventions. Solutions must address institutional inefficiencies and communication gaps, while also engaging with the deeper socio-economic and cultural dynamics that drive financial precarity. Through the lenses of vulnerability and social safety theories, it becomes clear that achieving equitable healthcare access requires systemic healthcare reform alongside broader socio-economic transformation.

### 5.5 Strengths of the study

This study draws on the theoretical frameworks of vulnerability and social safety to critically examine how institutional structures, cultural norms, and interpersonal relationships intersect to shape the production and experience of medical debt and hospital detention. These frameworks highlight how systemic inequalities, social expectations, and individual coping strategies are embedded within broader socio-political and economic arrangements, rather than arising in isolation.

A major strength of this research lies in its multi-perspectival and grounded approach. By incorporating the voices of detained patients, their families, and a wide range of healthcare providers—including nurses, billing clerks, social workers, and hospital administrators—the study captures the nuanced interplay between structural forces and lived experiences. This inclusion provides a more holistic understanding of how medical debt and hospital detention are produced, rationalized, and contested in practice. The diversity of perspectives also allows for the exploration of systemic issues such as financial barriers, informational gaps, and cultural beliefs that contribute to patient indebtedness and prolonged detention.

The demographic diversity of participants further enhances the study’s analytical depth, revealing the complex interactions among institutional practices, socio-economic vulnerabilities, and cultural norms. This demographic and experiential range strengthens the study’s capacity to uncover localized meanings and mechanisms underpinning medical debt.

Another methodological strength is the integration of cultural affinity training for fieldworkers. This training enhanced the ethical and cultural sensitivity of the research process by equipping interviewers with contextual knowledge and interpersonal skills tailored to local norms and expectations. As a result, fieldworkers were better able to build trust, reduce social distance, and facilitate candid dialogue—particularly important given the sensitive nature of hospital detention and indebtedness. This approach contributed to the collection of richer, more reliable, and ethically grounded data.

The regional focus of the research, conducted in a specific area of Cameroon, allowed for in-depth, contextually embedded analysis. Cameroon’s significant geographic, linguistic, and administrative diversity—particularly between the Anglophone and Francophone zones—meant that local interpretations of financial responsibility, family obligation, and access to care varied considerably. While this specificity may limit the generalizability of the findings to other regions or countries, it underscores the importance of localized analysis when designing health policy or intervention. Regional variation in governance, healthcare infrastructure, and cultural expectations must be considered when addressing inequities in healthcare access and outcomes.

In sum, the study’s strengths lie in its robust theoretical grounding, its inclusion of diverse stakeholder perspectives, its culturally responsive methodology, and its regionally focused lens. These elements combine to offer a contextually rich and critically informed analysis of medical indebtedness and hospital detention. While the findings are not statistically generalizable, they provide vital insights for region-sensitive health reforms and contribute meaningfully to national and academic debates on healthcare equity and social protection in Cameroon.

### 5.6 Implications for future research

While this study offers in-depth qualitative insights into the socio-cultural and systemic drivers of medical indebtedness and hospital detention, it has several limitations that should be addressed in future research. Notably, the absence of data on the total number of hospital admissions during the study period restricts the ability to assess the prevalence and scale of hospital detention. Additionally, the lack of clinical information on the diagnoses of detained patients limits understanding of how specific medical conditions—and their associated costs—may influence the likelihood or duration of detention. To build a more comprehensive picture, future studies should adopt mixed-methods approaches that integrate quantitative indicators of vulnerability—such as household income, health insurance coverage, and the strength of social support networks—with qualitative narratives. Comparative research across healthcare systems can further illuminate how institutional structures shape patients’ financial risks. Moreover, examining the intersectionality of gender and age—especially among vulnerable groups like teenage mothers and the older adults—can help guide more targeted, equitable interventions. Combining the contextual depth of ethnographic inquiry with the generalizability of statistical analysis will be crucial for informing evidence-based health policy reforms.

## 6 Conclusion

This study illuminates the complex and multifaceted nature of medical indebtedness and hospital detention in Cameroon, framing them not as isolated financial incidents but as manifestations of deeper structural and systemic vulnerabilities. Drawing on qualitative, anthropological insights and guided by vulnerability theory and social safety theory, the research reveals how intersecting individual, institutional, and cultural dynamics expose patients to disproportionate health and financial risks. Widespread poverty, the lack of universal health coverage, weak social protection systems, and entrenched cultural norms collectively erode individuals’ capacity to manage illness and secure timely, affordable care.

Crucially, this study challenges the dominant narrative that views debt as the root cause of vulnerability. Instead, it argues that enduring socio-economic inequalities and fragmented support structures create the conditions under which medical debt and hospital detention are normalized. In particular, the case of the Fundong Health District demonstrates how community expectations, systemic financing gaps, and institutional weaknesses converge to perpetuate hospital detention as a coping mechanism for underfunded health facilities.

By integrating vulnerability theory with social safety theory, this research offers a holistic framework for understanding the structural production of medical debt. These theoretical lenses underscore the urgent need for comprehensive reform—not only in healthcare financing but also within the broader architecture of care and social protection. Reducing hospital detention will require multidimensional strategies that address economic barriers, foster institutional accountability, shift cultural perceptions, and strengthen safety nets for those most at risk.

Ultimately, the findings call for context-sensitive, equity-driven policy responses that uphold patient dignity while addressing both the financial and psychosocial dimensions of healthcare access. Building on this foundation, the following recommendations propose a roadmap for meaningful and sustainable change.

### 6.1 Recommendations

To effectively confront medical debt and hospital detention in Cameroon, a targeted, multifaceted policy approach is crucial—one that directly addresses systemic financial barriers, institutional weaknesses, and cultural norms revealed in this study. The following specific recommendations are informed by the realities of the Fundong Health District and similar context.

1. Abolish maternal user fees and reduce costs of cesarean sections: Eliminating user fees for maternal health services—including antenatal care, delivery, and particularly cesarean sections—will alleviate financial barriers for pregnant women, who represent a highly vulnerable population. Subsidizing or fully covering the costs of cesarean procedures through government funding or donor support will help prevent catastrophic expenses that frequently result in medical debt and hospital detention.
2. Expand and strengthen health insurance coverage for vulnerable populations: Scale up formal health insurance schemes with a focus on including marginalized groups such as women, rural communities, and low-income households. Insurance packages should comprehensively cover essential services, including emergency surgeries and chronic illness management, to mitigate out-of-pocket expenses that drive hospital detention.
3. Enhance and protect social assistance funds in hospitals: Strengthen oversight and transparency mechanisms to ensure that social assistance funds—designed to support indigent patients unable to pay—are effectively managed and insulated from misappropriation. Hospitals should institutionalize flexible payment options such as accepting payments in kind, deferred payments, or community-based solidarity contributions to reduce reliance on detention as a coercive measure.
4. Develop community-based financial literacy and support programs: Implement culturally sensitive financial education initiatives that empower patients and families to better navigate healthcare costs, understand insurance benefits, and plan for potential medical expenses. These programs should also work to rebuild and reinforce informal social safety nets weakened by poverty and social fragmentation.
5. Reform hospital detention policies toward humane alternatives: Replace punitive hospital detention practices with policies that uphold patient dignity, including legal protections against unlawful detention and mechanisms for timely mediation of payment disputes. Establish dedicated social workers or patient advocates within hospitals to negotiate realistic payment plans and link patients to community resources.
6. Invest in mental health and psychosocial support services: Integrate mental health care into routine hospital services to address the psychological distress associated with medical debt and detention. Counseling and psychosocial support can help patients cope with trauma and reduce stigma, promoting holistic recovery.
7. Strengthen health system financing and governance: Address broader systemic gaps by increasing government investment in healthcare infrastructure, staffing, and supplies to reduce reliance on user fees. Improve regulatory frameworks and enforcement to ensure accountability in health financing and service delivery, thereby fostering trust in public health institutions.

By implementing these interconnected and contextually tailored recommendations, policymakers can begin to dismantle the structural drivers of medical debt and hospital detention. This will pave the way toward a more equitable, accessible, and compassionate healthcare system that protects the most vulnerable and respects the dignity of all patients.

## Data Availability

The raw data supporting the conclusions of this article will be made available by the authors, without undue reservation.
