# Supplementary material for: Trapped by debt: an ethnographic study of medical indebtedness and hospital detention in the Fundong Health District, Cameroon
Source: Front Public Health. 2025 Aug 26;13:1602798. doi: 10.3389/fpubh.2025.1602798 (PMC12417424; doi:10.3389/fpubh.2025.1602798)
Supplement: Supplementary file 1 [file Table_1.docx]

**Table 1:**

*Analysis of 100 Questionnaires on type of medicine purchased by clients and for whom*

| Reason | % | Self | Healers | Infant | Family | other | Patent | Herbal/  animal artefacts |
| --- | --- | --- | --- | --- | --- | --- | --- | --- |
| Malaria | 22 | 6 |  | 5 | 8 | 3 | 5 | 17 |
| lumbago | 15 | 10 |  |  |  |  | 5 | 10 |
| Concoction | 15 | 8 | 4 | 1 |  | 1 | 2 | 13 |
| Impotence | 8 | 6 |  |  |  | 2 | 1 | 7 |
| Anaemia | 8 | 3 |  | 5 |  |  | 4 | 4 |
| Chest pains | 8 | 5 | 2 | 1 |  |  | 2 | 6 |
| Venereal disease | 6 | 4 | 1 |  | 1 |  | 2 | 4 |
| Protection | 7 | 2 | 4 | 1 |  |  |  | 7 |
| Luck | 7 | 3 | 4 |  |  |  |  | 7 |
| Did not know | 4 |  |  |  | 2 | 2 | 1 | 3 |
| Totals (%) | 100% | 45 | 15 | 13 | 11 | 8 | 22 | 78 |

**^^[[1]](#footnote-1)^^**

**Table 2:**

*Plant species sold in African chemist as identified by Mr. Nsomfong Ndzana, National Haberium, Yaounde*

| Plant species | No. of botanical families |
| --- | --- |
| Hexalobus Crispflorus | 6 |
| Baillonella toxisperma | 7 |
| Erythropleum suaveollens | 8 |
| Annonaceue | 4 |
| mimosacea | 3 |
| Caesalpiniaceae | 3 |
| Euphorbiaceac | 3 |
| Total | 35 |

^^[[2]](#footnote-2)^^

1. Source: Fieldwork [↑](#footnote-ref-1)
2. Source: Sample collected during fieldwork [↑](#footnote-ref-2)
